# Comparative Analysis of Polyphenols in *Lycium barbarum* Fruits Using UPLC-IM-QTOF-MS

**DOI:** 10.3390/molecules28134930

**Published:** 2023-06-22

**Authors:** Yanjun Ju, Yujie Wang, Lei Ma, Lu Kang, Hejiang Liu, Xue Ma, Duoyong Zhao

**Affiliations:** Key Laboratory of Agro-Products Quality and Safety of Xinjiang, Institute of Agricultural Quality Standards and Testing Technology, Xinjiang Academy of Agricultural Sciences, Urumqi 830091, China; jyj1370@163.com (Y.J.); wyjxsz@163.com (Y.W.); mal0416@163.com (L.M.); 96208zx@163.com (L.K.); liuhejiang2025@163.com (H.L.); mx1838418725@163.com (X.M.)

**Keywords:** *Lycium barbarum*, polyphenols, UPLC-IM-QTOF-MS, antioxidant activity, variety, geographical origin

## Abstract

Variety, geographical origin, and harvest season are important factors affecting the accumulation of polyphenols in *Lycium barbarum*. In this study, the effects of these factors on the polyphenolic components of this species were analyzed using ultra-performance liquid chromatography ion mobility quadrupole time-of-flight mass spectrometry. Moreover, the in vitro antioxidant activities of fruit extracts from this species were evaluated. The total polyphenolic contents of *L. barbarum* fruits from Jinghe County in Xinjiang and Zhongning County in Ningxia were 5.52–11.72 and 7.06–9.37 mg (gallic acid equivalent)/g dry weight, while the total flavonoid contents of *L. barbarum* fruits from these regions were 12.52–30.29 and 12.67–20.77 mg (rutin equivalent)/g dry weight, respectively. Overall, 39 types of polyphenols were identified in the fruit extracts, including 26 flavonoids, 10 phenolic acids, and three tannins. Of these, 11 polyphenols were quantitatively analyzed, which revealed rutin to be the most dominant polyphenolic component in fruits from Jinghe and Zhongning. There were significant differences (*p* < 0.05) in the polyphenolic contents and antioxidant activities of *L. barbarum* fruit extracts, depending on the geographical origin, variety, and harvest season. The antioxidant activity of this species was found to be significantly positively correlated with the polyphenolic contents. This study provided scientific guidance for comprehensive applications of polyphenols from different varieties of *L. barbarum* from separate geographical origins.

## 1. Introduction

*Lycium barbarum* (Chinese wolfberry, Barbary wolfberry, or Chinese boxthorn) from the genus *Lycium* L. (*Solanaceae*) has received increased attention in recent years due to its traditional uses in Chinese herbal medicine and is listed in the “Pharmacopoeia of the People’s Republic of China” [1,2]. *L. barbarum* fruits are considered functional foods that have a large variety of beneficial effects. Recent studies have indicated that extracts of *L. barbarum* fruits possess a wide range of biological activities, including effects on aging, neuroprotection, anti-fatigue/endurance, anti-cancer activity, antioxidant properties, etc. [3,4]. At present, the species is distributed mainly in North and Northwest China, where it is widely cultivated as a high-yielding crop in the Qinghai, Ningxia, Gansu, Xinjiang, and Tianjin regions. *L. barbarum* fruit (wolfberry) is economically important and exported to more than 40 countries and regions. Moreover, Jinghe wolfberry and Zhongning wolfberry are listed in the China–EU Agreement on Cooperation and Protection of Geographical Indications [5]. Recently, *L. barbarum* fruits have become increasingly popular around the world because they contain diverse functional components, including amino acids, alkaloids, anthocyanins, polysaccharides, tocopherols, proteins, polyphenols, carotenoids, ascorbic acid, etc. [6,7,8,9,10,11,12].

Polyphenolic compounds, found abundantly in *L. barbarum* fruits, are secondary metabolites containing phenolic hydroxyl groups and include phenolic acids, flavonoids, and anthocyanins [13]. Polyphenols are known as the “seventh nutrient” for human health and have received much attention from researchers, due to their multifarious pharmacological properties, including hypoglycemic activity [14], antihyperlipidemic effects [15], activity in increasing metabolism [16,17], as well as antioxidant and anti-cancer properties [18,19,20,21]. Zhu et al. reported that phenolic amides extracted from *L. barbarum* fruits had better potential immunomodulatory activity than polysaccharides from this species and discovered three new phenolic amides and 12 analogues [22]. In a study of Goji berries, Bondia-Pons et al. identified 31 phenolic compounds and derivatives, including quercetin, coumaric acid, and caffeic acid and its derivatives, using non-targeted liquid chromatography coupled with quadrupole time-of-flight mass spectrometry (LC-qTOF-MS) [23]. Increasing the CO_2_ concentration and appropriately raising the temperature has been shown to contribute to the accumulation of polyphenols in onions [24]. Although researchers have conducted many studies on the contents and factors that influence polyphenols in *L. barbarum*, questions such as what further factors influence the accumulation of polyphenols in the fruits remain.

Ecological conditions and tillage methods have a significant impact on the quality and bioactive constituents of fruit. High temperature, moderate soil moisture, strong sunlight, and low altitude are suitable conditions for cultivating *L. barbarum* fruits with a high content of nutritious metabolites [25,26,27]. Lu et al. found significant differences in the nutritional components and antioxidant activities of *L. barbarum* from different regions in China [28]. Furthermore, Nzeuwa et al. reported slight differences in the functional components of *Lycium* fruits from different areas, demonstrating that the phenolic contents of *Lycium* fruits from Nepal were higher than in those from China [29]. Liu et al. discriminated between the nutritional components of *L. barbarum* from different geographical origins and varieties using nuclear magnetic resonance (NMR) techniques, finding that the variety Ningqi 9 had more nutritional value than the variety Ningqi 7 [30]. Cheng et al. found that black wolfberries from different geographical origins could clearly be identified by their individual anthocyanin contents detected using ultra-high-performance liquid chromatography (UPLC)-TOF-MS [31]. Therefore, geographical conditions greatly affect the accumulation of polyphenols. Differences in the main nutrients and phytochemicals present in *L. barbarum* from different geographical origins have been widely reported, but many studies have not been specific about the variety sampled or have been inconsistent in the maturation, cultivation, and management strategies of the *L. barbarum* samples studied. Little is known about the antioxidant activities and overall differences in the polyphenolic components of *L. barbarum* fruits from different areas and varieties.

Ultra-performance liquid chromatography ion mobility quadrupole time-of-flight mass spectrometry (UPLC-IM-QTOF-MS) is a recently developed, qualitative and quantitative technique for analyzing active ingredients in natural products and has been widely used to identify plant polyphenol compounds. One of the benefits of this technique is that it can be used to analyze the structure of components in extracts without the requirement for reference materials. UPLC-IM-QTOF-MS has the advantages of high resolution, high sensitivity, and accurate molecular weight analysis and can be used to predict the molecular formulas of compounds using accurate mass numbers and ion fragments, which enables the accurate identification of polyphenolic structures.

In this study, *L. barbarum* fruits grown in Jinghe, Xinjiang Province and Zhongning, Ningxia Province were selected as research objects. The total polyphenolic and flavonoid contents of the fruits were determined using the Folin–Ciocalteau method and aluminum trichloride colorimetry. Thereafter, differences in the polyphenolic components of different varieties of *L. barbarum* fruits grown in two geographical regions and harvested during two seasons were compared using UPLC-IM-QTOF-MS. This study provided useful information for quality improvement and product development strategies.

## 2. Results and Discussion

### 2.1. The Polyphenolic and Flavonoid Contents of L. barbarum Fruits from Different Geographical Origins

**Figure 1:**
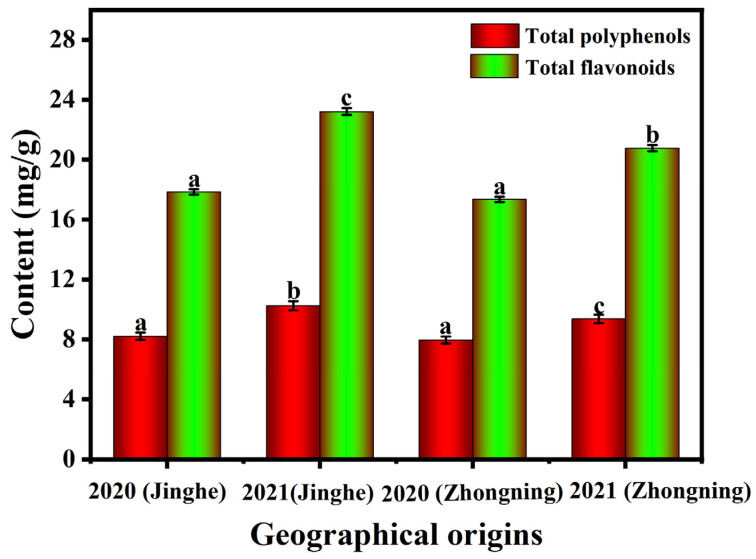
A comparison of the total polyphenolic and flavonoid contents of *L. barbarum* fruits harvested in summer 2020 and 2021 from different geographical origins. Different letters (a, b, c) across treatments indicate significant differences at *p* < 0.05.

The total polyphenolic and flavonoid contents of *L. barbarum* fruits (varieties 5# and 7#) harvested during summer 2020 and 2021 from Jinghe and Zhongning are shown in Figure 1. The total polyphenolic and flavonoid contents of the samples ranged from 5.52 to 11.72 mg gallic acid equivalent (GAE)/g dry weight (DW) and 12.52 to 30.29 mg GAE/g DW, respectively. The total polyphenolic and flavonoid contents of the two varieties grown in Jinghe were generally higher than of those grown in Zhongning, even though both varieties originated from the Zhongning area. There was a significant difference (*p* < 0.05) between the polyphenolic contents of *L. barbarum* fruits harvested from the two regions in 2021. To explore the impact of meteorological factors on the accumulation of polyphenols in *L. barbarum*, the corresponding meteorological data for the samples grown in different geographical origins were analyzed statistically (Table S1). Altitude was taken as the average for all sample collection sites, and other meteorological data were obtained by the China Meteorological Administration. Temperature, difference in temperature, extreme temperature, annual precipitation, altitude, and hours of sunlight were recorded for the whole of the year in 2020. Comparing the meteorological conditions in Jinghe and Zhongning in 2020, Jinghe had a longer duration of sunlight, lower precipitation, and lower mean temperature, which might have been more conducive to the accumulation of polyphenolic compounds. In addition, Jinghe is surrounded by mountains on all sides, experiences drought with little rain, and is rich in sandy and saline–alkali soil, all of which are conducive to the healthy growth of *L. barbarum*.

### 2.2. The Polyphenolic and Flavonoid Contents in Four Varieties of L. barbarum

The total polyphenolic and flavonoid contents of *L. barbarum* samples from four varieties (5#, 7#, 9#, and 1801#) grown in Jinghe were measured according to the Folin–Ciocalteau method, and the results are shown in Table 1 and Table S2. In fruits harvested during 2020 and 2021, the total polyphenolic and flavonoid contents in 7# and 1801# were the highest, followed by those in 5# and 9#. The highest contents of total polyphenols and flavonoids were 11.72 and 30.29 GAE/g DW, respectively. There were significant differences among the total polyphenolic contents of the four varieties (*p* < 0.05). Among them, the differences seen in 1801# and 9# in different harvesting periods were more significant. The variation in the total flavonoid contents of the different varieties was similar to that of the total polyphenolic contents. Comprehensive analysis showed that the polyphenolic content of 7# was the highest and that of 9# harvested in summer was the lowest.

### 2.3. The Polyphenolic Contents of L. barbarum Harvested during Different Seasons

Analyzing the total polyphenolic contents of *L. barbarum* revealed that fruits harvested in autumn had significantly higher levels than those harvested in summer (Table 1 and Table S2). Summer-harvested *L. barbarum* fruits are generally collected as four crops, while the autumn fruits are collected as two to three crops; thus, the ripening cycle of the autumn *L. barbarum* fruits is longer, and the temperature difference between day and night in autumn is relatively larger than that in summer, so the nutrients can fully accumulate in the fruits. Moreover, the polyphenolic contents of fruits collected in 2021 were obviously higher than those in 2020, which might have been caused by differences in the climate, sunlight duration, and temperature.

### 2.4. Comparison of Antioxidant Activity

#### 2.4.1. Antioxidant Activity of *L. barbarum* Fruits Harvested from Different Geographical Origins

**Figure 2:**
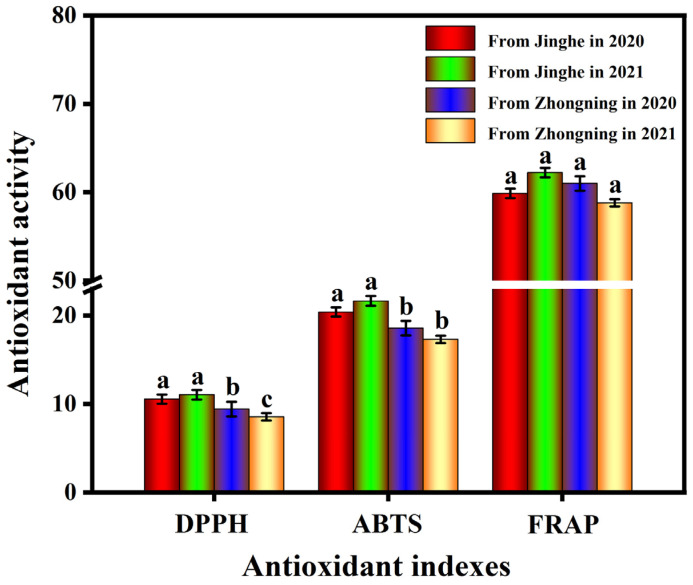
The antioxidant activity of *L. barbarum* fruits grown in different geographical origins. Different letters (a, b, c) across treatments indicate significant differences at *p* < 0.05.

The antioxidant activity of *L. barbarum* fruits harvested from Jinghe and Zhongning in 2020 and 2021 was analyzed by measuring the DPPH (2,2-diphenyl-1-picrylhydrazyl) and ABTS (2,2′-azino-bis(3-ethylbenzothiazoline-6-sulfonic acid)) radical scavenging capacity and the ferric ion reducing antioxidant potential (FRAP). The DPPH method has good reproducibility due to its long free-radical half-life. The ABTS method can be used to measure the antioxidant capacity of hydrophilic and lipophilic substances; the reduced ABTS radical is colorless due to a color-quenching reaction. The principle of the FRAP method is that, with the antioxidant at low pH, ferric tripyridyltriazine (Fe(III)-TPTZ) is reduced to ferrous tripyridyltriazine (Fe(II)-TPTZ) and can be measured at 597 nm, accompanied by the appearance of blue color. The FRAP method can inhibit some endogenous interference factors. Therefore, the above three methods are widely used to evaluate the antioxidant activity of plant extracts. As shown in Figure 2, the antioxidant activity of *L. barbarum* extracts from fruits grown in different geographical origins had different capacities. The DPPH and ABTS radical scavenging and FRAP results from fruits grown in Jinghe were significantly higher than those in fruits grown in Zhongning in 2021 (*p* < 0.05). The DPPH and ABTS values of *L. barbarum* harvested from Jinghe were higher than those from Zhongning in 2020, but the FRAP value of *L. barbarum* harvested from Zhongning was higher than that in fruits harvested from Jinghe.

#### 2.4.2. Antioxidant Activity of Fruit Extracts from Different Varieties of *L. barbarum*

The antioxidant activity (DPPH, ABTS, and FRAP) of *L. barbarum* fruit extracts from varieties 5#, 7#, 9#, and 1801#, harvested from Jinghe, was measured. Table 2 shows that the antioxidant activities among the four varieties were significantly different (*p* < 0.05). In particular, 7# showed the highest DPPH radical scavenging ability and FRAP, with values of 12.09 mg Vc/g DW and 63.76 mmol Vc/g DW, respectively, while the ABTS radical scavenging ability of 7# was slightly lower than that of 1801#, with a value of 22.41 mmol Vc/g DW. The comprehensive analysis revealed that the antioxidant activities of 7# and 1801# were significantly higher than those of 5# and 9#. The order of antioxidant capacity in the different varieties was similar to that of the total polyphenolic and total flavonoid contents.

The relationship between the total polyphenolic content, total flavonoid content, and antioxidant activity (DPPH, ABTS, and FRAP) of *L. barbarum* fruit extracts was evaluated using correlation analysis. As shown in Table S3, the total polyphenolic content was extremely significantly correlated with DPPH radical scavenging ability and FRAP (*p* < 0.01) and was significantly correlated with ABTS radical scavenging ability (*p* < 0.05). The correlation between total flavonoids and antioxidant activity (DPPH, ABTS, and FRAP) was extremely significant (*p* < 0.01). The results revealed that the amount of total polyphenols and total flavonoids had an important influence on the antioxidant activity of *L. barbarum* fruit extracts, which is consistent with a previous report [32].

Based on previous work and the results obtained from the present study, polyphenols are likely to be the important active compounds present in *L. barbarum*. A prior analysis of the antioxidant activity of phenolic acids and flavonoids in *L. barbarum* indicated that the flavonoid components such as rutin and quercetin were important for scavenging DPPH free radicals [33]. In addition, the abilities of *L. barbarum* to scavenge and prevent the formation of free radicals are closely related to the concentration and composition of the polyphenols. Thus, it was speculated that polyphenols may be the key active components contributing to the antioxidant activity of *L. barbarum*. The reason for the differences in antioxidant activity (DPPH, ABTS, and FRAP) of different varieties of *L. barbarum* and those from different regions might be due to the diversity of the polyphenols. The types and contents of polyphenolic compounds with antioxidant effects in *L. barbarum* showed certain differences in antioxidant activities.

### 2.5. Analysis of Polyphenolic Compounds in L. barbarum Fruit Extracts

The metabolomics method of UPLC-IM-QTOF-MS was established for the non-targeted qualitative and quantitative analysis of polyphenolic compounds in *L. barbarum* fruit extracts. UPLC-IM-QTOF-MS non-targeted screening showed a wide and chemically diverse profile of polyphenolic compounds in comparison to previous knowledge.

#### 2.5.1. Qualitative Analysis of Polyphenols in *L. barbarum* Fruit Extract

**Figure 3:**
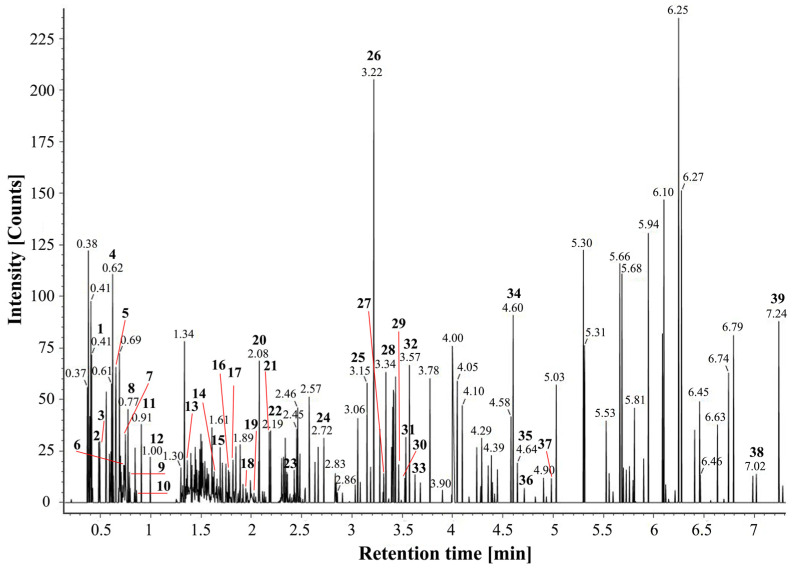
Total ion chromatogram of *L. barbarum* fruit extract in positive-ion mode (Numbers 1–39 represent different characteristic peaks recognized).

A total ion chromatogram for an *L. barbarum* fruit extract in positive-ion MS^E^ mode is shown in Figure 3. This provided a relatively integrated picture of the metabolomic analysis and formed an analytical fingerprint for the identification and authentication of fruits or fruit-derived products. The identified polyphenols mainly peaked within 0.40–7.25 min. The exact *m*/*z* ratio, retention time, error, and fragment ions of characteristic peaks were shown in Table S4. By comparing the Waters Progenesis QI database (including natural product library, metabolite library, sugar metabolism library, and endogenous compound library) and related reports [6,34,35,36], a total of 39 polyphenolic compounds were identified in the *L. barbarum* fruit extract, including 26 flavonoids, 10 phenolic acids, and three tannins.

**Figure 4:**
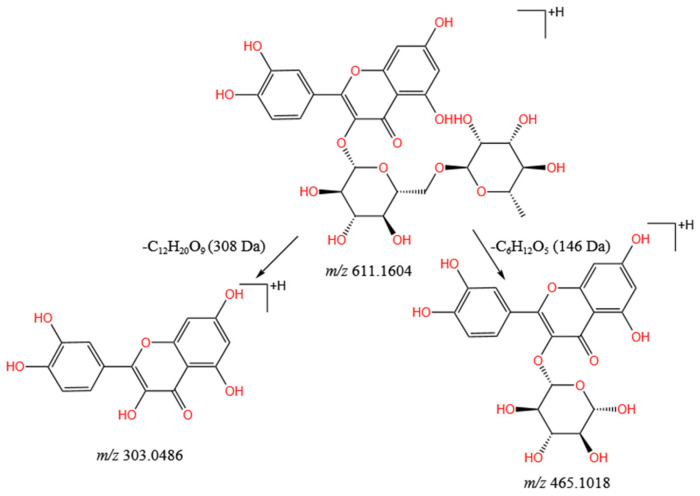
Main cleavage pathway of rutin.

Flavonoids consists of three ring skeletons, C_6_-C_3_-C_6_, which are derived from the parent nucleus of flavone (2-phenylchromone) [37]. Twenty-six flavonoids were identified accurately in the *L. barbarum* fruit extract by comparing the Waters Progenesis QI database and previous literature reports, including flavanones (compound **15**), flavones (compound **9**), flavonols (compounds **2**, **18**, **21**, **22**, and **25**–**32**), chalcone (compound **35**), flavanes (compounds **7**, **16**, **17**, and **33**), and anthocyanidins (compounds **1**, **3**, **10**, **13**, **24**, **36**, and **37**). Taking compound **25** in Table S4 as an example, the main cracking mechanism of this compound was analyzed. The retention time of compound **25** was 3.22 min, and it had a protonated molecular ion [M + H]^+^ at *m*/*z* 611.1604. Compound **25** generated MS/MS characteristic ions at *m*/*z* 465.1018 (isoquercetin [M + H]^+^) and at *m*/*z* 303.0486 (quercetin [M + H]^+^), because *m*/*z* 611.1604[M + H]^+^ lost 146 Da (rhamnoside) and 308 Da (rutinoside) [38]. The main cleavage pathway is shown in Figure 4. By comparing the retention time and MS/MS fragment information with the rutin reference substance, compound **25** was presumed to be rutin. This speculation was consistent with previous reports [39,40].

**Figure 5:**
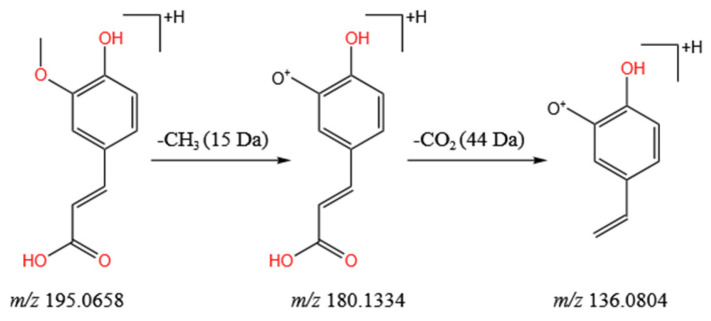
Main cleavage pathway of ferulic acid.

Phenolic acids are compounds with phenolic groups and organic carboxylic acid functional groups, and most of them are hydroxybenzoic acid derivatives with a C_6_-C_1_ skeleton and hydroxycinnamic acid derivatives with a C_6_-C_3_ skeleton [41,42]. They have potential biological properties such as antioxidation, antiviral, antibacterial, and anti-inflammatory activities [43]. A total of 11 phenolic acids were accurately identified in the *L. barbarum* fruit extract, including hydroxybenzoic acids (compounds **6**, **11**, **12**, and **38**) and hydroxycinnamic acids (compounds **4**, **8**, **14**, **20**, **23**, and **34**). Taking compound **20** in Table S4 as an example, the main cracking process of phenolic acid compounds was analyzed. The retention time of compound **20** was 2.08 min. By comparing MS/MS fragment information with the rutin reference substance and related literature [44], compound **20** with *m*/*z* at 195.0658 [M + H]^+^ was determined as ferulic acid, because it showed characteristic MS/MS ions at *m*/*z* 180.1334 [M−CH_3_ + H]^+^ and *m*/*z* 136.0804 [M−CH_3_−CO_2_ + H]^+^ (Figure 5).

**Figure 6:**
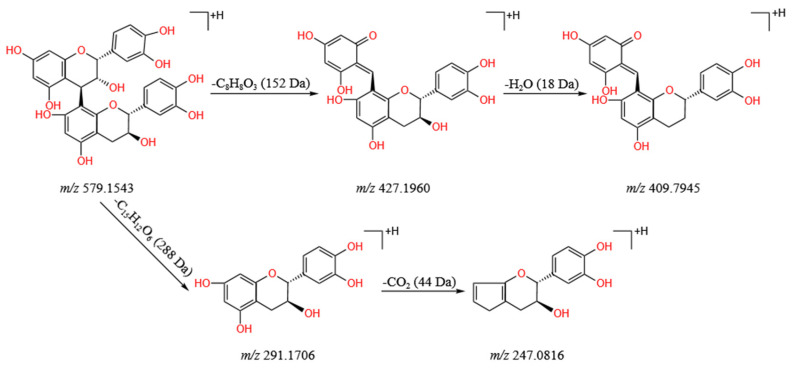
Main cleavage pathway of procyanidin B_1_.

Tannins are water-soluble polyphenols with a relative molecular mass of between 500 and 3000, which exist widely in plants. Three tannins (compounds **5**, **19**, and **39**) were accurately identified in the *L. barbarum* fruit extract. Taking compound **19** in Table S4 as an example, the main cracking mechanism of tannins was analyzed. The retention time of compound **19** was 2.03 min, and its protonated molecular ion was at *m*/*z* 579.1534 [M + H]^+^. As shown in Figure 6, compound **19** showed fragment ions at *m*/*z* 427.1960 [M−C_8_H_8_O_3_ + H]^+^; *m*/*z* 409.7945 (loss of C_8_H_8_O_3_ and H_2_O, respectively); *m*/*z* 291.1706 [M−288 + H]^+^; and 247.0816 [M−288−CO_2_ + H]^+^. By comparing the retention time, the Waters Progenesis QI database, and a previous report [45], compound **19** was inferred to be procyanidin B_1_.

#### 2.5.2. Quantitative Analysis of Polyphenols in *L. barbarum* Fruit Extract

**Figure 7:**
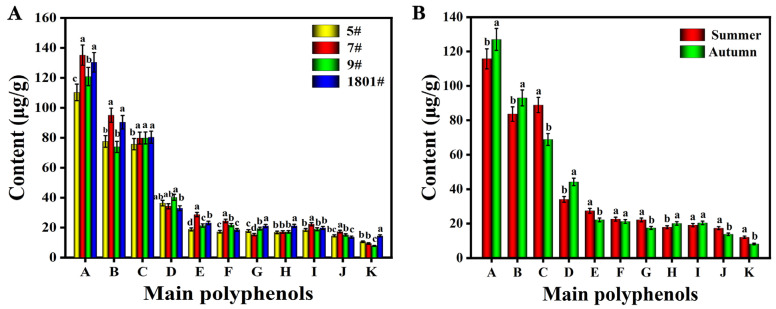
Comparison of the main polyphenolic contents of fruits from different varieties of *L. barbarum* (**A**) harvested during different seasons (**B**). (A, rutin; B, *p*-coumaric acid; C, ferulic acid; D, quercetin; E, kaempferol-3-*O*-rutinoside; F, isoquercetin; G, chlorogenic acid; H, isorhamnetin-3-*O*-rutinoside; I, isorhamnetin-3-glucoside; J, isorhamnetin; K, caffeic acid). Different letters across treatments (a, b, c, d) indicate significant differences at *p* < 0.05.

Based on the results of the qualitative analysis, the 11 main polyphenols in the *L. barbarum* fruit extract were quantitatively analyzed using the UPLC-IMS-QTOF-MS targeted metabolomics method and the external standard method. As shown in Table 3, the contents of rutin and *p*-coumaric acid were highest in the extract from *L. barbarum* fruit grown in Jinghe, at 125.38 μg/g and 90.05 μg/g, respectively, while the content of caffeic acid was the lowest, with a value of 10.44 μg/g. Most of the polyphenolic compounds in *L. barbarum* from Jinghe were found at higher levels than in fruit from Zhongning. In comparing varieties and harvest seasons (Figure 7), the contents of the main polyphenolic compounds in 7# and 1801# were generally higher than those in 5# and 9#, and the contents of rutin and *p*-coumaric acid found in autumn-harvested fruits were significantly higher than those in summer-harvested fruits, while ferulic acid was the opposite. The above analysis revealed that the polyphenolic compounds in fruits from different varieties of *L. barbarum* harvested in different seasons showed obvious differences, which provide favorable data for further targeted selection of *L. barbarum* for research purposes.

## 3. Materials and Methods

### 3.1. Chemicals and Reagents

Leucine enkephalin was purchased from Waters Corporation (Milford, MA, USA). Chromatography-grade methanol, mass-spectrometry-grade acetonitrile, and formic acid were purchased from Thermo Fisher Scientific (Waltham, MA, USA). Folin & Ciocalteu phenol reagent was bought from Sigma-Aldrich, Co., Ltd. (St. Louis, MO, USA). Ferrous sulfate, ferric chloride, and analytical-grade ethyl alcohol were procured from Tianjin Xinbo Chemical Co., Ltd. (Tianjin, China). Analytical-grade anhydrous sodium carbonate (Na_2_CO_3_), aluminum chloride (AlCl_3_), potassium acetate, and gallic acid monohydrate (purity 99.0%) were obtained from Tianjin Yongsheng Fine Chemical Co., Ltd. (Tianjin, China). The phenolic standards such as rutin, quercetin, ferulic acid, caffeic acid, isorhamnetin, kaempferol 3-*O*-beta-rutinoside, *p*-coumaric acid, chlorogenic acid, and isoquercetin were purchased from Anpel Laboratory Technologies Inc. (Shanghai, China). Hydrochloric acid was purchased from Xi’an Chemical Reagent Factory (Xi’an, China). The 2,2′-azino-bis (3-ethylbenzothiazoline-6-sulfonic acid) (ABTS), 1,1-diphenyl-2-picrylhydrazyl (DPPH), 2,4,6-tris (2-pyridyl)-*s*-triazine (TPTZ), and sodium acetate were provided by Beijing Solarbio Science & Technology Co., Ltd. (Beijing, China). Ultrapure water was purchased from Watsons Group Ltd. (Hong Kong, China).

### 3.2. Instruments

The polyphenolic compounds were determined with an ultra-performance liquid chromatography ion mobility quadrupole time-of-flight mass spectrometer (UPLC-IM QTOF-MS) (Waters, Milford, MA, USA). The absorbance was measured using an ultraviolet and visible spectrophotometer (UV-2700) for the determination of total polyphenols and total flavonoids (Shimadzu, Kyoto, Japan). An XSE 204 balance was used to weigh *L. barbarum* samples (Mettler-Toledo, Greinfesee, Switzerland). The samples were mixed using an MS3 vortex mixer (IKA, Staufen, Germany) and centrifuged using a Sorvall biofuge Stratos system (Thermo Fisher Scientific, Waltham, MA, USA). The *L. barbarum* extracting solution was concentrated using a Hei-VAP Precision rotary evaporator (Heidolph, Schwabach, Germany).

### 3.3. Sample Collection

**Figure 8:**
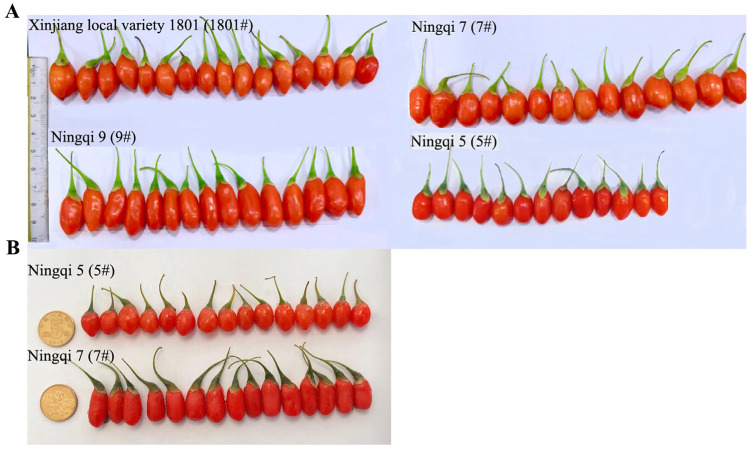
(**A**) Four varieties (1801#, 7#, 9#, and 5#) of fresh *L. barbarum* fruits from Jinghe Xinjiang and (**B**) two varieties (5# and 7#) of fresh *L. barbarum* fruits from Zhongning.

Four varieties of fresh *L. barbarum* fruit samples were collected from Jinghe County, Boertala Mongol Autonomous Prefecture, Xinjiang (E 82°17′–83°51′, N 44°32′–45°10′; altitude: 270–390 m) and Zhongning County, Zhongwei City, NingXia (E 105°33′–106°22′, N 37°17′–38°49′; altitude: 1080–1372 m) from June to September in 2020 and 2021, respectively, including “Ningqi 5#” (5#), “Ningqi 7#” (7#), “Ningqi 9#” (9#), and “Xinjiang local variety 1801#” (1801#) collected from Jinghe County (Figure 8A) and “Ningqi 5#” and “Ningqi 7#” collected from Zhongning County (Figure 8B). The *L. barbarum* fruit samples were at the same stage of maturity, with complete fruit grains and no diseases or pests. The fresh *L. barbarum* fruits were stored at −20 °C for further use. Overall, 150 samples were collected.

### 3.4. Extract Collection

The fresh *L. barbarum* fruits were freeze-dried in a vacuum, ground into powder, and stored in brown glass bottles at −4 °C. Briefly, the dry ground *L. barbarum* samples (1.0 g) were added to 20 mL of 70% methanol solution containing 1.0% hydrochloric acid in 50 mL plastic centrifuge tubes. After mixing thoroughly, the samples were ultrasonicated at 40 °C for 30 min and then centrifuged at 10,000 rpm for 10 min using a high-speed refrigerated centrifuge. The supernatant was collected, and the extraction process was repeated twice. Then, the supernatants were combined and filtered. The mixture was adjusted to 35 mL with methanol and stored at −4 °C for further experiment.

### 3.5. Measurement of Total Polyphenols

The concentration of total polyphenols was detected using the Folin–Ciocalteau method according to a previous report with some modifications [46]. Gallic acid was regarded as an equivalent weight. The data were expressed as mg gallic acid equivalents (GAE)/g of dry weight (DW). Absorbance was measured at 765 nm. As shown in Figure S1, the content of total polyphenols was measured according to a standard calibration curve (y = 0.8861x + 0.0151) with a linear range from 0 to 1.75 mg/mL (R^2^ = 0.9997).

### 3.6. Measurement of Total Flavonoids

The concentration of total flavonoids was determined according to an aluminum trichloride colorimetric method, as reported by Wu et al. [47]. Rutin was used as the equivalent weight. The total flavonoid content was expressed as mg rutin equivalent (RE)/g of DW. Absorbance was measured at 415 nm. The content of total flavonoids was measured according to the standard calibration curve (y = 0.2823x + 0.0274) with a linear range from 0 to 4 mg/mL (R^2^ = 0.9997) (Figure S2).

### 3.7. Antioxidant Activity Analysis

#### 3.7.1. Determination of DPPH Radical Scavenging Ability

The DPPH radical scavenging ability was determined using a DPPH radical scavenging capacity assay kit. Absorbance was measured with a multiscan spectrum microplate spectrophotometer (Bio Tek, Winooski, VT, USA) at 515 nm. First, different concentrations of positive control vitamin C (V_C_) standard solution (0.01–0.3 mg/mL) were prepared. The standard calibration curve was obtained (y = 0.7421x + 0.1428, R^2^ = 0.9922) by calculating the DPPH radical scavenging rate of V_C_. The DPPH radical scavenging ability of each *L. barbarum* sample was calculated, and the results were expressed as V_C_ equivalent per gram of *L. barbarum* sample (mg V_C_/g DW). The radical scavenging rates of the positive control (1) and DPPH (2) were calculated as follows.

Radical scavenging rate of positive control (%) = [(A_0_ − A_control_)/A_0_] × 100 (1)

Radical scavenging rate of DPPH (%) = [A_0_ − (A_1_ − A_2_)/A_0_] × 100 (2)

where A_0_ is the absorbance of the blank solution; A_control_ is the absorbance of V_C_; A_1_ is the absorbance of the added sample; and A_2_ is the absorbance of the sample control.

#### 3.7.2. Determination of ABTS Radical Scavenging Ability

The ABTS radical scavenging ability was measured using an ABTS radical scavenging capacity assay kit. Absorbance was measured with a multiscan spectrum microplate spectrophotometer (Bio Tek, Winooski, VT, USA) at 405 nm. Different concentrations of positive-control V_C_ standard solution (0.1–1 mmol/L) were prepared. The standard calibration curve was obtained (y = 0.7045x − 0.0076, R^2^ = 0.9962) by calculating the ABTS radical scavenging rate of V_C_. The ABTS radical scavenging ability was expressed as V_C_ equivalents per gram of *L. barbarum* sample (mmol V_C_/g DW). The formulae used to calculate the radical scavenging rate of the positive control and ABTS were the same as in Section 3.7.1.

#### 3.7.3. Determination of Ferric Ion Reducing Antioxidant Power

The FRAP was determined based on a previously reported method with some modifications [48]. FeSO_4_ standard solutions were prepared at different concentrations (0.5−4.0 mmol/mL). The FRAP working solutions consisted of 10 mmol/L TPTZ solution, 0.3 mol/L sodium acetate (pH 3.6), and 20 mmol/L FeCl_3_ solution in the ratio of 1:10:1. The mixture was placed in a water bath at 35 °C for 30 min. Then, 0.1 mL of FeSO_4_ standard solution and 0.9 mL of FRAP working solution were added to 6 mL of ultrapure water. After mixing them well and placing in a water bath at 35 °C for 30 min, the absorbance was measured at 597 nm. The standard calibration curve was obtained as y = 0.1356x + 0.0899 (R^2^ = 0.9994). The FRAP value was expressed as Fe^2+^ equivalents per gram of *L. barbarum* sample (mmol Fe^2+^/g DW).

### 3.8. UPLC-IM-QTOF-MS Analysis

UPLC-IM-QTOF-MS was used to detect the phenolic compounds in the *L. barbarum* samples. Firstly, the *L. barbarum* fruit extract was filtered through a 0.22 μm filtration membrane and injected into the UPLC-IM-QTOF-MS system. The polyphenolic compounds in the prepared samples were separated in an ACQUITY UPLC-BEN C18 column (2.1 × 50 mm, 1.7 μm; Waters, Milford, MA, USA) at 35 °C. The mobile phase was water (A) and acetonitrile (B). The gradient elution was as follows: 0−1 min, 99% A; 1−10 min, A from 99% to 0%; 10−11 min, 0% A; and 11−13 min, A from 0% to 99%. The flow rate was 0.3 mL/min, and the injection volume was 5 μL.

Ion source: electrospray ionization (ESI) was used in positive-ion mode with the following parameters: source temperature, 100 °C; capillary voltage, 3 kV; desolvation temperature, 350 °C; gas flow, 900 L/h; low collision energy, 6 eV; and higher collision energy, 10 to 40 eV.

### 3.9. Statistical Analysis

All data are presented as the mean ± standard deviation. SPSS 26.0 software was used to perform statistical analysis. Significant differences in the polyphenol concentrations in *L. barbarum* fruits from different geographical origins and varieties were calculated using a one-way ANOVA, along with the Duncan test. UNIFI 1.8 software and the Waters Progenesis QI database were used for qualitative and quantitative analysis of polyphenolic compounds in *L. barbarum*. Origin 2019b software was used to compile charts.

## 4. Conclusions

The total polyphenol and flavonoid contents of different *L. barbarum* fruit varieties grown in two geographical origins, Jinghe and Zhongning, and harvested during different seasons were analyzed. The polyphenol contents of *L. barbarum* fruits harvested in autumn were generally higher than those of fruits harvested in summer. The polyphenolic contents and antioxidant capacities (DPPH, ABTS, and FRAP) of fruits from different geographical origins and varieties of *L. barbarum* were significantly different (*p* < 0.05). A UPLC-IM-QTOF-MS metabolomics-based method was established for the qualitative and quantitative analysis of the polyphenolic compounds in *L. barbarum*. A total of 39 polyphenolic compounds were identified, including 26 flavonoids, 10 phenolic acids, and three tannins. It could be seen that the polyphenols in Jinghe-sourced *L. barbarum* were mainly flavonoids, followed by phenolic acids. The contents of all kinds of polyphenols of *L. barbarum* grown in Jinghe were generally higher than in those harvested from Zhongning, which revealed that more hours of sunlight and an appropriate amount of water shortage were more conducive to the accumulation of polyphenols. In addition, the contents of polyphenolic compounds in different varieties of *L. barbarum* and in fruits harvested during different seasons showed obvious differences. This study explored the polyphenolic components of *L. barbarum*, providing scientific and technological support for further high-value development and utilization of *L. barbarum*.

## Figures and Tables

**Table 1:** The total polyphenolic contents of different varieties of *L. barbarum* harvested during different seasons (X ± s, *n* = 3).

Variety	Contents in the Summer of 2020 (mg GAE/g DW)	Contents in the Autumn of 2020 (mg GAE/g DW)	Contents in the Summer of 2021 (mg GAE/g DW)	Contents in the Autumn of 2021 (mg GAE/g DW)
5#	7.64 ± 0.24 ^b^	9.35 ± 0.29 ^c^	9.18 ± 0.88 ^bc^	9.63 ± 0.37 ^c^
7#	8.21 ± 0.40 ^a^	10.88 ± 0.66 ^a^	10.25 ± 0.60 ^a^	10.08 ± 0.59 ^b^
9#	5.52 ± 0.22 ^c^	9.54 ± 0.27 ^c^	8.70 ± 0.98 ^c^	9.90 ± 0.53 ^bc^
1801#	8.14 ± 0.48 ^a^	10.24 ± 0.13 ^b^	9.70 ± 0.82 ^b^	11.72 ± 0.60 ^a^

Different lowercase letters (a, b, c) per column represent significant differences (*p* < 0.05).

**Table 2:** Antioxidant activity (DPPH, ABTS, and FRAP) of different *L. barbarum* (X ± s, *n* = 3).

Variety	DPPH (mg V_C_/g DW)	ABTS (mmol V_C_/g DW)	FRAP (mmol V_C_/g DW)
5#	10.68 ± 1.10 ^b^	20.81 ± 0.63 ^bc^	59.15 ± 1.72 ^c^
7#	12.09 ± 1.03 ^a^	21.76 ± 1.04 ^ab^	63.76 ± 1.91 ^a^
9#	9.43 ± 1.38 ^c^	19.97 ± 1.80 ^c^	59.87 ± 2.01 ^c^
1801#	11.60 ± 0.71 ^a^	22.41 ± 1.23 ^a^	61.43 ± 2.72 ^b^

Different lowercase letters (a, b, c) per column represent significant differences (*p* < 0.05).

**Table 3:** Main polyphenolic contents of *L. barbarum* fruits harvested from different geographical origins (*n* = 50).

Compounds	Linear Equation	Linear Range (μg/L)	R^2^	Contents in Jinghe (μg/g)	Contents in Zhongning (μg/g)
Rutin	y = 16.283x + 205.61	1500–90,000	0.9978	125.38 ± 21.89	98.59 ± 23.76
*p*-coumaric acid	y = 8.9186x − 15058	3300–12,000	0.9973	90.05 ± 11.46	70.38 ± 6.97
Ferulic acid	y = 3.7786x − 5304	5000–20,000	0.9957	78.75 ± 3.41	62.01 ± 5.65
Quercetin	y = 12.129x − 21.878	450–6500	0.9954	38.62 ± 3.19	41.32 ± 8.23
Kaempferol-3-*O*-rutinoside	y = 29.865x + 732.33	30–1500	0.9957	25.12 ± 3.60	18.22 ± 3.12
Isoquercetin	y = 18.515x + 802.05	100–900	0.9946	21.54 ± 2.98	13.39 ± 2.40
Isorhamnetin-3-glucoside	y = 28.776x + 586.64	10–180	0.9929	21.13 ± 2.82	17.56 ± 2.53
Chlorogenic acid	y = 4.7343x − 767.2	400–3600	0.9960	18.65 ± 2.80	10.06 ± 2.71
Isorhamnetin-3-*O*-rutinoside	y = 31.045x + 672.94	40–360	0.9962	18.17 ± 1.20	15.34 ± 1.91
Isorhamnetin	y = 32.993x + 487.01	50–450	0.9939	15.30 ± 2.17	11.59 ± 3.61
Caffeic acid	y = 1.3820x − 1179.7	10,000–70,000	0.9931	10.44 ± 2.62	9.06 ± 2.15

## Data Availability

Data available on request from the authors.

## Supplementary Materials

The following supporting information can be downloaded from: https://www.mdpi.com/article/10.3390/molecules28134930/s1. Table S1: The climatic conditions of the different geographical origins at which L. barbarum samples grew in 2020; Table S2: The total flavonoid contents of different *L. barbarum* varieties harvested during summer and autumn (X ± s, *n* = 3); Table S3: Correlation of total polyphenolic content, total flavonoid content, and antioxidant capacity of *L. barbarum*; Table S4: The polyphenolic compounds identified in *L. barbarum;* Figure S1: The regression equation of gallic acid; Figure S2: The regression equation of rutin.

## Sample Availability

Samples of *L. barbarum* used in this study are available from the authors.
