# Supplementary material for: Comparative Analysis of Polyphenols in Lycium barbarum Fruits Using UPLC-IM-QTOF-MS
Source: Molecules. 2023 Jun 22;28(13):4930. doi: 10.3390/molecules28134930 (PMC10343790; doi:10.3390/molecules28134930)
Supplement: Supplementary file 1 [file molecules-28-04930-s001.zip › molecules-2411001-supplementary.pdf]

Supplementary Material

Comparative Analysis of Polyphenols in *Lycium barbarum* Fruits Using UPLC-IM-QTOF-MS

Yanjun Ju, Yujie Wang, Lei Ma, Lu Kang, Hejiang Liu, Xue Ma and Duoyong Zhao *

Key Laboratory of Agro-Products Quality and Safety of Xinjiang, Institute of Agricultural Quality Standards and Testing Technology, Xinjiang Academy of Agricultural Sciences, Urumqi 830091, China; jyj1370@163.com (Y.J.); wyjxsz@163.com (Y.W.); mal0416@163.com (L.M.); 96208zx@163.com (L.K.); liuhejiang2025@163.com (H.L.); mx1838418725@163.com (X.M.)

* Correspondence: luckydyz@163.com; Tel.: +86-0991-4541-379

Table S1. The climatic conditions of the different geographical origins at which *L. barbarum* samples grew in 2020.

Geographical origins	Altitude (m)	MT (°C)	MTD (°C)	Sunlight hours (h)	MT in Jan (°C)	MT in July (°C)	EHT (°C)	ELT (°C)	Annual precipitation (mm)
Jinghe	310	9.1	10.4	2927	-14.9	28.0	42.6	-24.9	106.8
Zhongning	1290	11.4	12.6	2673	-5.4	24.7	36.9	-14.2	226.1

MT: Mean temperature, MTD: Mean temperature difference, MT in Jan: Mean temperature in January, EMT: Extreme highest temperature, ELT: Extreme lowest temperature.

Table S2. The total flavonoid contents of different *L. barbarum* varieties harvested during summer and autumn (X±s, n= 3).

Variety	Contents in the summer of 2020 (mg GAE/g DW)	Contents in the autumn of 2020 (mg GAE/g DW)	Contents in the summer of 2021 (mg GAE/g DW)	Contents in the autumn of 2021 (mg GAE/g DW)
5#	13.74±0.95[b]	22.76±2.53[b]	18.91±1.72[bc]	25.43±1.62[c]
7#	17.85±1.55[a]	25.67±0.99[a]	23.22±1.83[a]	28.51±1.58[b]
9#	12.52±1.72[b]	24.21±1.58[ab]	18.27±0.79[c]	27.71±1.15[b]
1801#	16.55±1.48[a]	23.45±1.24[b]	20.11±2.08[b]	30.29±1.82[a]

Different lowercase letters per column represent significant differences ($P < 0.05$).

Table S3. Correlation of total polyphenolic content, total flavonoid content and antioxidant capacity of *L. barbarum.*

Antioxidant activity	Correlation coefficient(r)	
	**Total polyphenolic content**	**Total flavonoid content**
DPPH	0.969**	0.945**
ABTS	0.752*	0.914**
FRAP	0.866**	0.809**

*Correlation is significant ($P < 0.05$).

**Correlation is extremely significant ($P < 0.01$).

Table S4. The polyphenolic compounds identified in *L. barbarum.*

No	Rt (min)	Theoretical (m/z)	Observed (m/z)	Error (mDa)	Formula	Fragmentation (m/z)	Response	Compound
1	0.41	331.0818	331.0790[M+H]$^+$	-2.80	$C_{17}H_{15}O_7$	182.9747; 270.9460	3988	Malvidin
2	0.48	434.0849	452.1192[M+NH$_4$]$^+$	2.80	$C_{20}H_{18}O_{11}$	303.1009	2511	Quercetin-3-D-xyloside
3	0.49	493.1346	511.1659[M+NH$_4$]$^+$	-2.50	$C_{23}H_{25}O_{12}$	333.1073	3880	Malvidin-3-glucoside
4	0.62	224.0685	242.1022[M+NH$_4$]$^+$	-1.10	$C_{11}H_{12}O_5$	209.0649; 165.0598	14226	Sinapic acid
5	0.65	198.0528	216.0864[M+NH$_4$]$^+$	–0.40	$C_9H_{10}O_5$	171.0345; 126.9910; 101.01286	13795	Ethyl gallate
6	0.73	122.0368	123.0442[M+H]$^+$	0.10	$C_7H_6O_2$	79.0384	5606	Benzoic acid
7	0.75	306.0740	308.0818[M+H]$^+$	0.50	$C_{15}H_{14}O_7$	249.0765	12447	Gallocatechin
8	0.77	164.0473	182.0811[M+NH$_4$]$^+$	-1.30	$C_9H_8O_3$	149.0638; 121.0489; 93.0541	10707	p-coumaric acid
9	0.80	596.1741	635.1395[M+Na]$^+$	3.10	$C_{27}H_{32}O_{15}$	419.3364; 289.9804; 165.0767	3395	Eriocitrin
10	0.86	287.0556	288.0652[M+H]$^+$	2.70	$C_{15}H_{11}O_6$	271.1600; 215.1321	4163	Cyanidin
11	0.91	138.0317	156.0656[M+NH$_4$]$^+$	0.10	$C_7H_6O_3$	94.8913; 66.9245	4345	p-hydroxybenzoicacid
12	1.00	168.0423	186.0758[M+Na]$^+$	-0.50	$C_8H_8O_4$	154.0565; 125.0442	5966	Vanillic acid
13	1.36	463.1240	464.1307[M+H]$^+$	-0.70	$C_{22}H_{23}O_{11}$	303.0907	7429	Peonidin--3-O-glucoside
14	1.63	180.0422	198.0763[M+NH$_4$]$^+$	-0.40	$C_9H_8O_4$	147.0437	4157	Caffeic acid
15	1.69	610.1898	628.2238[M+NH$_4$]$^+$	1.50	$C_{28}H_{34}O_{15}$	303,02738; 288.03627	60547	Hesperidin
16	1.78	290.0790	291.0838[M+H]$^+$	-2.50	$C_{15}H_{14}O_6$	273.1837; 247.0728; 229.0178	9277	Catechin
17	1.82	458.0849	497.0487[M+K]$^+$	0.70	$C_{22}H_{18}O_{11}$	291.0274; 247.0275	2656	Epigallocatechin gallate
18	1.95	478.1111	501.0999[M+Na]$^+$	-0.40	$C_{22}H_{22}O_{12}$	319.0652; 304.0411	15529	Isorhamnetin-3-glucoside
19	2.03	578.1424	579.1534[M+H]$^+$	3.7	$C_{30}H_{26}O_{12}$	429.1960; 411.7945; 293.1706; 249.0816	2104	Procyanidin B_1
20	2.08	194.0579	195.0658[M+H]$^+$	-1.40	$C_{10}H_{10}O_4$	180.1334; 136.0804	6749	Ferulic acid
21	2.18	304.0583	322.0928[M+NH$_4$]$^+$	0.40	$C_{15}H_{12}O_7$	166.0420; 109.0281	1892	Dihydroquercetin
22	2.19	450.1162	473.1041[M+Na]$^+$	-1.30	$C_{21}H_{22}O_{11}$	153.0752; 304.1217	1284	Dihydroquercetin-3-o-rhamnoside
23	2.43	354.0951	355.1027[M+H]$^+$	0.50	$C_{16}H_{18}O_9$	91.0386; 119.0333; 165.0388; 180.1227	5804	Chlorogenic acid
24	2.72	508.1217	509.1272[M+H]$^+$	-3.50	$C_{23}H_{24}O_{13}$	329.0617; 347.0674	11845	Syringetin-3-galactoside

No	Rt (min)	Theoretical (m/z)	Observed (m/z)	Error (mDa)	Formula	Fragmentation (m/z)	Response	Compound
25	3.15	302.0427	303.0501[M+H]$^+$	0.50	$C_{15}H_{10}O_7$	275.1674; 259.5721; 181.1372	15191	Quercetin
26	3.22	610.1534	611.1604[M+H]$^+$	0.40	$C_{27}H_{30}O_{16}$	465.1018; 303.0486	94200	Rutin
27	3.32	302.0427	303.0499[M+H]$^+$	-0.90	$C_{15}H_{10}O_7$	231.1618; 139.0726	13652	Morin
28	3.34	464.0955	465.1035[M+H]$^+$	0.50	$C_{21}H_{20}O_{12}$	303.0962	13883	Isoquercetin
29	3.46	594.1585	595.1673[M+H]$^+$	1.60	$C_{27}H_{30}O_{15}$	451.1085; 287.0976	37065	kaempferol-3-o-rutinoside
30	3.52	448.1006	449.1087[M+H]$^+$	0.30	$C_{21}H_{20}O_{11}$	205.0367; 289.0563	9215	kaempferol-3-o-glucoside
31	3.54	624.1690	625.1765[M+H]$^+$	0.50	$C_{28}H_{32}O_{16}$	319.0674	12366	Isorhamnetin-3-o-rutinoside
32	3.57	316.0583	317.0634[M+H]$^+$	-2.10	$C_{16}H_{12}O_7$	302.1746; 153.2847	10176	Isorhamnetin
33	3.63	442.0900	460.1235[M+NH$_4$]$^+$	-0.40	$C_{22}H_{18}O_{10}$	186.1338; 111.0282	6588	Catechin-3-o-gallate
34	4.60	148.0524	149.0601[M+H]$^+$	-1.10	$C_9H_8O_2$	132.0647; 104.0271	22706	Cinnamic acid
35	4.64	436.1370	437.1417[M+H]$^+$	-2.60	$C_{21}H_{24}O_{10}$	109.0363; 277.0464; 169.0365	4200	Phlorizin
36	4.82	479.1190	518.0825[M+K]$^+$	-3.67	$C_{22}H_{23}O_{12}$	319.0372	1079	Petunidin-3-o-glucoside
37	4.98	611.1612	612.1702[M+H]$^+$	-2.40	$C_{27}H_{31}O_{16}$	499.036; 289.0365	2785	Cyanidin-3,5-o-diglucoside
38	7.02	211.0481	212.0534[M+H]$^+$	-1.70	$C_9H_9NO_5$	110.0365; 82.0648	1548	Gentisuric acid
39	7.24	634.0806	657.0758[M+Na]$^+$	5.60	$C_{27}H_{22}O_{18}$	303.0637; 277.0274	16021	Corilagin

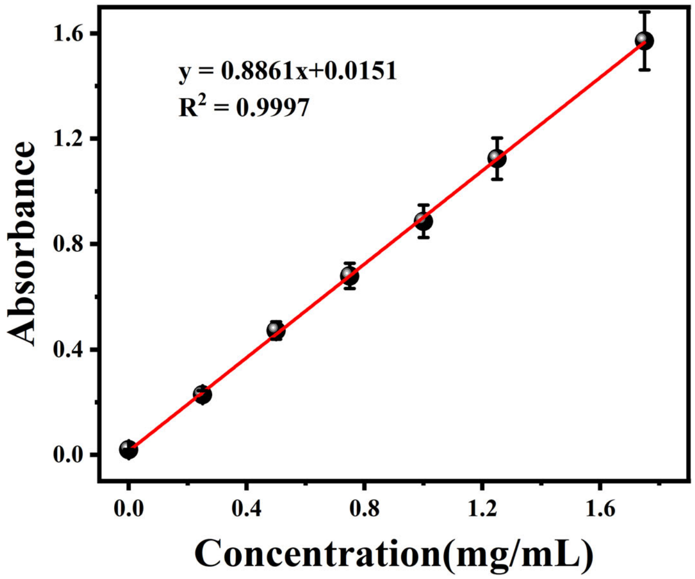

Figure S1. The regression equation of gallic acid.

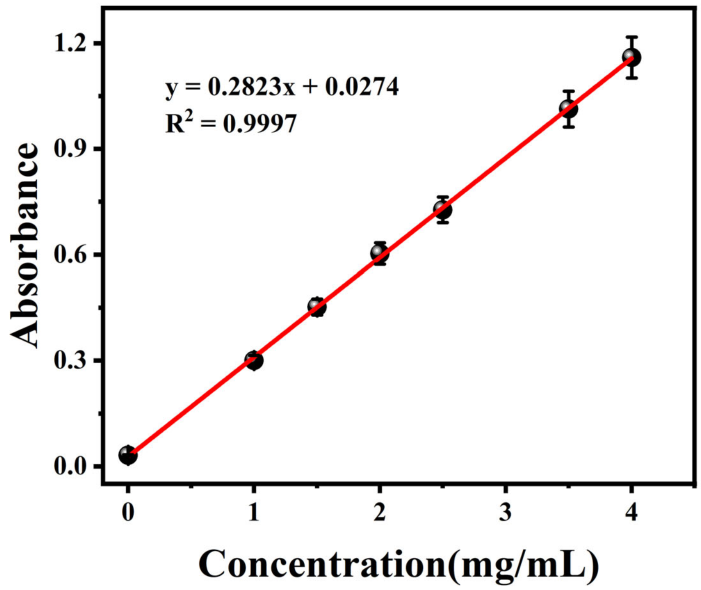

Figure S2. The regression equation of rutin.
